# 

**Published:** 1886-12-18

**Authors:** 


PRICE ONE PENNY.

No. 12, Vol. I.

December 18th, 1886.

Per Annum, 6s. 6d., post free.
Monthly Parts, 6d.; post free, 7½d.
Ditto per Ann., 7s. 6d., post free.

